# Evaluation of serum vitamin D metabolites, phagocytosis, and biomarkers of inflammation in dogs with naturally occurring diabetes mellitus

**DOI:** 10.3389/fvets.2024.1441993

**Published:** 2024-08-21

**Authors:** Jared A. Jaffey, Robert C. Backus, Rachael Kreisler, Thomas K. Graves, Layla Al-Nakkash, Lauren Allison

**Affiliations:** ^1^Department of Specialty Medicine, College of Veterinary Medicine, Midwestern University, Glendale, AZ, United States; ^2^Department of Veterinary Medicine and Surgery, College of Veterinary Medicine, University of Missouri, Columbia, MO, United States; ^3^Department of Pathology and Population Medicine, College of Veterinary Medicine, Midwestern University, Glendale, AZ, United States; ^4^Department of Physiology, College of Graduate Studies, Midwestern University, Glendale, AZ,United States

**Keywords:** 25(OH)D, 25-hydroxyvitamin D, C-reactive protein, cytokines, glycemic control

## Abstract

Naturally occurring diabetes mellitus (NODM) is one of the most common endocrine disorders in dogs and its etiology closely resembles type 1 diabetes mellitus (T1DM) in people. Human patients with T1DM commonly have cellular derangements consistent with inflammation, impaired immune function, and hypovitaminosis D. There is little information available regarding inflammatory biomarkers, immune function, and vitamin D status in diabetic dogs. Therefore, our objectives were to assess inflammatory biomarkers, vitamin D metabolites, and phagocytic capacity in diabetic dogs and determine whether associations exist with these variables and the level of clinical control or vitamin D metabolites. This was a prospective case–control study that included 20 otherwise healthy diabetic dogs (clinically controlled, *n* = 10; uncontrolled, *n* = 10) and 20 non-diabetic, healthy, age (± 2 years), breed, and sex matched controls. Complete blood count, biochemical panel, urinalysis, and fructosamine were performed at a single commercial reference laboratory. Basal plasma tumor necrosis factor (TNF)-α, interleukin (IL)-6, IL-8, and IL-10 were measured using a canine-specific multiplex bead-based assay. Serum C-reactive protein (CRP) was measured using a commercially available ELISA kit. Serum 25-hydroxyvitamin (OH)D_3_ and 24,25-dihydroxyvitamin (OH)_2_D_3_ were measured with HPLC. Phagocytosis of opsonized-*Escherichia coli* (*E. coli*) was evaluated with flow cytometry. Diabetic dogs had higher serum CRP concentrations than controls (*p* = 0.02). Plasma IL-8 concentrations were higher in diabetic dogs with uncontrolled clinical disease compared to controls (*p* = 0.02). Diabetic dogs had a lower percentage of leukocytes that phagocytized opsonized-*E. coli* (*p* = 0.02), but an increased number of bacteria phagocytized per cell (*p* < 0.001) compared to controls. No between-group differences were identified in vitamin D metabolites, nor were associations found between vitamin D and any variables. Fructosamine had a positive association with serum CRP concentration (rho = 0.35, *p* = 0.03) and number of bacteria phagocytized per cell (rho = 0.45, *p* = 0.004) in our cohort (*n* = 40). Like people with T1DM, diabetic dogs have a proinflammatory phenotype and phagocytic dysregulation that may be correlated with glycemic control.

## Introduction

Diabetes mellitus is one of the most common endocrine disorders in middle-to older-aged dogs with a prevalence in the pet population worldwide that ranges from 0.2 to 1.3% (1–9). Diabetes mellitus is characterized by persistent hyperglycemia because of reduced pancreatic β-cell secretion of insulin, insulin sensitivity in peripheral tissues, or both. The most commonly recognized form of naturally occurring diabetes mellitus (NODM) in dogs is similar to type 1 diabetes mellitus (T1DM) in people and is characterized by hypoinsulinemia and a dependence on exogenous insulin administration to maintain euglycemia, mitigate ketosis, and survive (10). Common clinical signs demonstrated by diabetic dogs include one or more of polyuria, polydipsia, polyphagia, and weight loss (11).

Diabetic complications in human patients are related to protracted hyperglycemia and resultant downstream effects which contribute to cytokine dysregulation characterized by a proinflammatory state and immune dysfunction (12–20). Furthermore, proinflammatory cytokines directly and indirectly contribute to insulin resistance that negatively affects glycemic control in people with T1DM (17, 21, 22). Therefore, it is intuitive that proinflammatory biomarkers, including C-reactive protein (CRP) and cytokines, have been shown to be useful adjunctive tools to assess glycemic control and predict risk for diabetic related complications in human patients (16, 23, 24). There is a fraction of available information regarding the inflammatory state and immune function in dogs with NODM despite similar risks for infectious and inflammatory disorders and the development of insulin resistance (25–28). Expanding our knowledge of the inflammatory milieu and immune function in diabetic dogs will improve our understanding of this endocrinopathy.

Numerous studies have demonstrated a link between T1DM in people with hypovitaminosis D and direct comparisons of absolute blood 25-hydroxyvitamin (OH)D concentrations are consistently lower than non-diabetic controls (29–31). Several studies have shown that hypovitaminosis D at different life stages is associated with the eventual development of T1DM in human patients (32–35). These results suggest that vitamin D status may be considered an environmental risk factor for T1DM in people, especially at some life stages. However, hypovitaminosis D in some diabetic patients may be multifactorial or exclusively a result of the diabetic syndrome rather than causal. Regardless of whether vitamin D is a contributory factor or consequence, understanding the vitamin D status in diabetic people has proven useful because of its association with glycemic control and development of complications (36–38). Moreover, vitamin D supplementation in people with T1DM decreases proinflammatory biomarkers and improves glycemic control, although conflicting reports exist (39–41).

This prospective case–control study had three objectives (i) to compare granulocyte/monocyte (GM) phagocytic capacity of *Escherichia coli* (*E. coli*), concentrations of serum vitamin D metabolites, CRP, and basal plasma cytokines in diabetic dogs and non-diabetic healthy controls, (ii) assess whether clinical control of diabetes mellitus affects these variables, and (iii) evaluate whether associations exist between vitamin D metabolites and inflammatory biomarkers or phagocytic capacity. We hypothesized that diabetic dogs would have higher inflammatory biomarkers, phagocytic dysregulation, and altered vitamin D metabolites compared to controls and differences would be magnified in diabetic dogs with uncontrolled clinical signs. In addition, we hypothesized that one or more vitamin D metabolites would be associated with ≥1 of serum CRP, plasma cytokine, or phagocytic capacity of *E. coli*.

## Materials and methods

### Animals and study design

Client-owned dogs with NODM that were otherwise healthy and treated with ≥0.25 units/kg of insulin administered once every 12 h were eligible for inclusion. Diagnosis of diabetes mellitus was performed according to criteria outlined in the 2018 AAHA Diabetes Management for Dogs and Cats (11). Specifically, the presence of hyperglycemia, glucosuria, and ≥ 1 related clinical sign (e.g., polyuria, polydipsia, polyphagia, and weight loss). Diabetic dogs were classified as having clinically controlled diabetes mellitus if the dog exhibited no polyuria, polydipsia, polyphagia, and there had been no dose adjustments to insulin within 4 weeks of enrollment. Therefore, dogs were considered to have uncontrolled diabetes mellitus if they exhibited ≥1 of related clinical signs without a rationale alternative cause. Exclusion criteria included obesity (i.e., body condition score (BCS) ≥ 6/9), vaccination within 1-month, concurrent illness within 2 months of enrollment, or presence of a clinically relevant comorbid disorder. A board-certified small animal internist (JAJ) determined whether a comorbid condition was clinically relevant. A second population of healthy, age (i.e., ± 2 years), breed, and sex-matched non-diabetic healthy control dogs were enrolled. Control dogs were included after review of clinical history, physical examination, complete blood count, urinalysis, and serum chemistry by a single investigator (JAJ). Control dogs were enrolled in the study if they met the following requirements: non-obese, no illnesses within 6 months of enrollment, and had not received any vaccinations within 1 month of enrollment. The study protocol was approved by the Midwestern University Animal Care and Use Committee (protocol #2944) with written owner consent. Dogs were enrolled from December 2019 to July 2020. Dogs included in this study were used in a separate study focused on the *ex vivo* effects of calcitriol on several immunological variables in diabetic dogs (42).

### Data and sample collection

Medical records were reviewed for each dog enrolled. The age, sex, weight, BCS, and breed were recorded. Other salient details from the medical records included: maintenance diet information, date of initial diabetes mellitus diagnosis, insulin type, and dose. Hematology, serum biochemistry, serum fructosamine, and urinalysis tests from the day of enrollment were measured at a commercial laboratory (Antech Diagnostics, United States).

Blood was collected from a jugular or peripheral vein and transferred into serum separator blood tubes and lithium heparin anticoagulated blood tubes. Blood from the lithium heparin anticoagulated blood tube was processed within 1 h of collection for experiments related to phagocytosis. The remaining lithium heparin anticoagulated blood was centrifuged, and plasma transferred to freezer-resistant conical microcentrifuge tubes and stored at −80°C for batch analysis of cytokines. Similarly, blood in the serum separator blood tubes was allowed to clot and then centrifuged with serum transferred to freezer-resistant conical microcentrifuge tubes and stored at −80°C for batch analysis of 25-hydroxyvitamin (OH)D_3_, 24,25-dihydroxyvitamin (OH)_2_D_3_, and CRP.

### Constitutive plasma cytokine concentrations

Plasma samples were thawed, after which tumor necrosis factor (TNF)-α, interleukin (IL)-6, IL-8, and IL-10 were quantified with a canine cytokine-specific multiplex bead-based assay (Milliplex MAP, EMD Millipore Corp) as previously described (43). The median fluorescence intensity and cytokine concentration in each sample was measured in duplicate with appropriate controls and associated data analysis software (Milliplex Analyst version 5.1, EMD Millipore Corp). The lower limit of detection for TNF-ɑ, IL-6, and IL-10 was 48.8 pg./mL and IL-8 was 195 pg./mL.

### C-reactive protein concentrations

Serum samples were thawed, and CRP was measured with a commercially available canine-specific ELISA (Abcam, Cambridge, United Kingdom) as previously described (44, 45). Samples were measured in duplicate with concurrent standard curves using kit-provided canine standards; the lower limit of detection was 1.1 ng/mL. The optical density of the samples was determined with a Biotek Cytation 3 microplate reader (Biotek, Vermont, United States) set to a wavelength of 450 nm. Background absorbance was measured at 700 nm and subtracted from sample absorbance. Sample CRP concentration was determined by plotting the kit standards using a linear curve.

### 25(OH)D_3_ and 24,25(OH)_2_D_3_ concentrations

Serum concentrations of 25(OH)D_3_ and 24,25(OH)_2_D_3_ were determined in thawed samples using previously reported HPLC methods (46). The lower limit of quantification of 25(OH)D_3_ and 24,25(OH)_2_D_3_ was 3.0 ng/mL and 9.1 ng/mL, respectively. The vitamin D metabolite ratio (VMR) has been suggested to be a superior surrogate marker for vitamin D status compared to 25(OH)D_3_ alone because it is not influenced by concentrations and genetic variants of vitamin D binding protein in people (47). The VMR is calculated with the following formula: 24,25(OH)_2_D_3_/25(OH)D_3._

### Phagocytosis of *E. coli*

Phagocytic capacity of GM was determined with a commercially available assay (PhagoTest, Orpegen Pharma), validated for use in canines. Heparinized blood was incubated with FITC-labelled, opsonized-*E. coli* strain LE392. The control samples were incubated on ice for 10 min while test samples were incubated in a 37°C water bath for 10 min (43). Phagocytosis was then arrested with samples placed on ice and a quenching solution was added to extinguish surface bound FITC-labelled-*E. coli*. The cells were then washed, erythrocytes lysed, and cells washed again before DNA staining (R-phycoerythrin) solution was added to facilitate exclusion of aggregated artifacts of bacteria or cells without intact DNA.

### Flow cytometry

Flow cytometry was performed at the Midwestern University College of Veterinary Medicine Immunology Laboratory using a flow cytometer (Guava easyCyte HT, Luminex Corporation) and associated data analysis software (GuavaSoft 3.2, Luminex Corporation). A minimum of 20,000 events/sample were recorded. Our gating scheme has previously been reported (43). For assessment of phagocytosis, data was recorded as the percentage of GM cells having internalized FITC-labelled *E. coli*, as well as their mean fluorescent intensity (MFI), a method of quantifying the phagocytosed bacteria per cell.

### Statistical analysis

Statistical analyses were performed using a commercial software (SigmaPlot, Systat Software version 14.5). Categorical data were presented as proportions. Normality was assessed using the Shapiro–Wilk test. Non-normally distributed data were presented as median, interquartile range (IQR), and range when indicated. Normally distributed data were presented as mean and standard deviation (SD). When the measured cytokine, CRP, 25(OH)D_3_, or 24,25(OH)_2_D_3_ concentration fell below the lower limit of detection, data were recorded at the lower limit of detection for statistical purposes. Student’s *t*-test was used for two group comparisons of normally distributed continuous variables, and Mann–Whitney rank sum test for non-normally distributed continuous variables. A Kruskal-Wallis One-Way Analysis of Variance (ANOVA) test, followed by a Dunn’s test for multiple comparisons was used for multi-group comparisons of non-normally distributed data while a One-Way ANOVA was used for normally distributed data. Spearman’s correlation coefficient was used to assess the strength of relationship between serum 25(OH)D_3_, 24,25(OH)_2_D_3_, and VMR with CRP, IL-8, and phagocytic function. As outlined below, TNF-α, IL-6, and IL-10 were below the lower limit of detection in most samples precluding their inclusion in statistical analyses. The strength level of the relationship between variables was defined with the following Spearman’s rho intervals: weak (0.1–0.39), moderate (0.4–0.69), strong (0.7–0.89), and very strong (0.9–1.0) (48). A *P*-value of < 0.05 was considered significant.

## Results

### Animals

Forty-one dogs were eligible for inclusion in this study. One dog was excluded because a matched non-diabetic control dog was not identified, leaving 40 dogs included in the study (NODM, *n* = 20; controls, *n* = 20). Demographic information for this cohort is published (42). The median duration of time from diagnosis of NODM to enrollment was 302.5 days (IQR, range; 363.25, 41–1,113 days). Diabetic dogs were treated with either neutral protamine Hagedorn (NPH) (55%, 11/20) or porcine Lente (45%, 9/20) insulin. The median dosage of insulin administered once every 12 h to diabetic dogs was 0.75 units/kg (IQR, range; 0.48, 0.3–1.2 units/kg). Dogs were fed a commercially available pet food (Supplementary material). Three control dogs were suspected to have mild osteoarthritis. These 3 dogs were administered a joint health supplement alone (*n* = 1) or in conjunction with gabapentin (*n* = 1) or gabapentin alone (*n* = 1). The joint health supplement administered to both dogs was Cosequin (Nutramax Laboratories, Veterinary Sciences, Inc). Gabapentin was administered to the two dogs as needed and was not given within 30 days of enrollment. None of the remaining dogs were administered supplements or medications.

Ten dogs each were clinically controlled or uncontrolled. Demographic information is summarized in Table 1. There were no differences in age, weight, BCS, insulin dosage, duration of time with NODM, serum fructosamine or glucose concentrations between diabetic dogs that were clinically controlled and those that were uncontrolled (*p* > 0.05; Table 1).

**Table 1:** Descriptive characteristics in dogs with naturally occurring diabetes mellitus (*n* = 20) that were clinically controlled (*n* = 10) or uncontrolled (*n* = 10).

Variable	Controlled	Uncontrolled	*p*-value
Age (years)^a^	9.7 (2.2)	8.9 (1.6)	0.41
Weight (kgs)^b^	6.4 (8.3)	7.9 (10.6)	0.55
BCS^b^	5 (1)	5 (1)	0.79
Sex (female, male)	5, 5	5, 5	–
Neutered (yes, no)	9, 1	10, 0	–
Breed	Chihuahua (*n* = 2), Labrador retriever-mix (*n* = 2), Bichon frise, Miniature pinscher, Pomeranian, Rottweiler, Poodle-mix, Havanese	Chihuahua (*n* = 2), Australian shepherd-mix, Labrador retriever, Jack Russel terrier-mix, Miniature pinscher-mix, Maltese-poodle mix, Rat terrier, Australian cattle dog, Yorkshire terrier	–
Insulin dosage (units/kg)^a^	0.67 (0.27)	0.70 (0.30)	0.82
Time with NODM (days)^b^	351 (355.3)	223 (397)	0.94
Fructosamine (μmol/L)^b^	519.5 (201.5)	458.5 (173)	0.45
Glucose (mg/dL)^a^	343.2 (165.6)	367.4 (236.2)	0.79

BCS, body condition score; kgs, kilograms; NODM, naturally occurring diabetes mellitus. ^a^Data presented as mean (standard deviation). ^b^Data presented as median (interquartile range).

### Basal plasma cytokines

**Figure 1:**
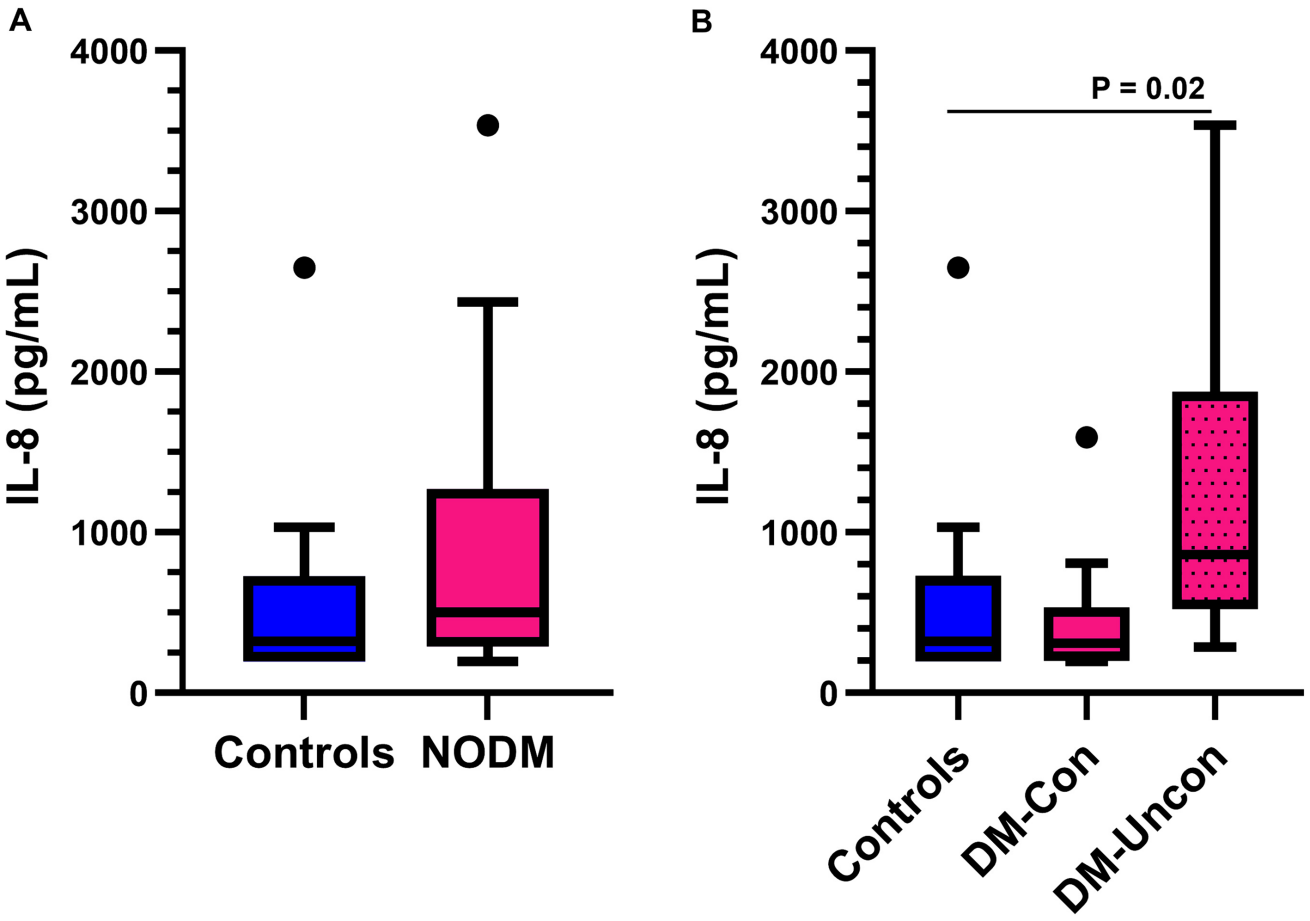
Box and whisker plots comparing basal plasma interleukin (IL)-8 concentrations between **(A)** dogs with naturally occurring diabetes mellitus (NODM) and controls, and between **(B)** diabetic dogs that were clinically controlled (DM-Con), uncontrolled (DM-Uncon), and controls. Line at median, bounds of box at the 25th and 75th percentile, whiskers at the upper and lower adjacent values (Tukey method), and dots at outliers beyond the adjacent values. **(A)** There was no difference in plasma IL-8 concentrations between diabetic dogs (median, IQR; 502.5 pg./mL, 981.4) and controls (323.2 pg./mL, 531.9, *p* = 0.09). **(B)** There was a difference between the medians for controls, DM-Con (307.3 pg./mL, 349.5, *n* = 10), and DM-Uncon (860.1 pg./mL, 1354.7, *n* = 10, *p* = 0.02). Plasma IL-8 concentrations were higher in DM-Uncon compared to controls (*p* = 0.02). No differences were found between DM-Con and controls (*p* = 1.00) or DM-Con and DM-Uncon (*p* = 0.06).

There was no difference in plasma IL-8 concentrations between diabetic dogs and controls (*p* = 0.10, Figure 1A). However, subgroup analysis revealed a difference between controls, and diabetic dogs that were clinically controlled and uncontrolled (*p* = 0.02). Diabetic dogs that were clinically uncontrolled had higher plasma IL-8 concentrations than controls (*p* = 0.02, Figure 1B).

Plasma TNF-α, IL-6, and IL-10 concentrations were below the limit of detection in most dogs, regardless of group. Specifically, TNF-α, IL-6, and IL-10 concentrations were below the lower limit of detection in 85% (34/40) (NODM, *n* = 19; controls, *n* = 15), 85% (34/40) (NODM, *n* = 19; controls, *n* = 15), and 98% (39/40) (NODM, *n* = 20; controls, *n* = 19) of dogs, respectively. The same 6 dogs had both quantifiable plasma TNF-α and IL-6 concentrations. The median TNF-α and IL-6 concentrations in these dogs was 131.4 pg./mL (IQR, 222) and 94.6 pg./mL (IQR, 133.2). The one dog with a quantifiable plasma IL-10 concentration (78.6 pg./mL) had TNF-α and IL-6 below the limit of detection. The lack of quantifiable results for these cytokines precluded group or multigroup comparisons.

### C-reactive protein

**Figure 2:**
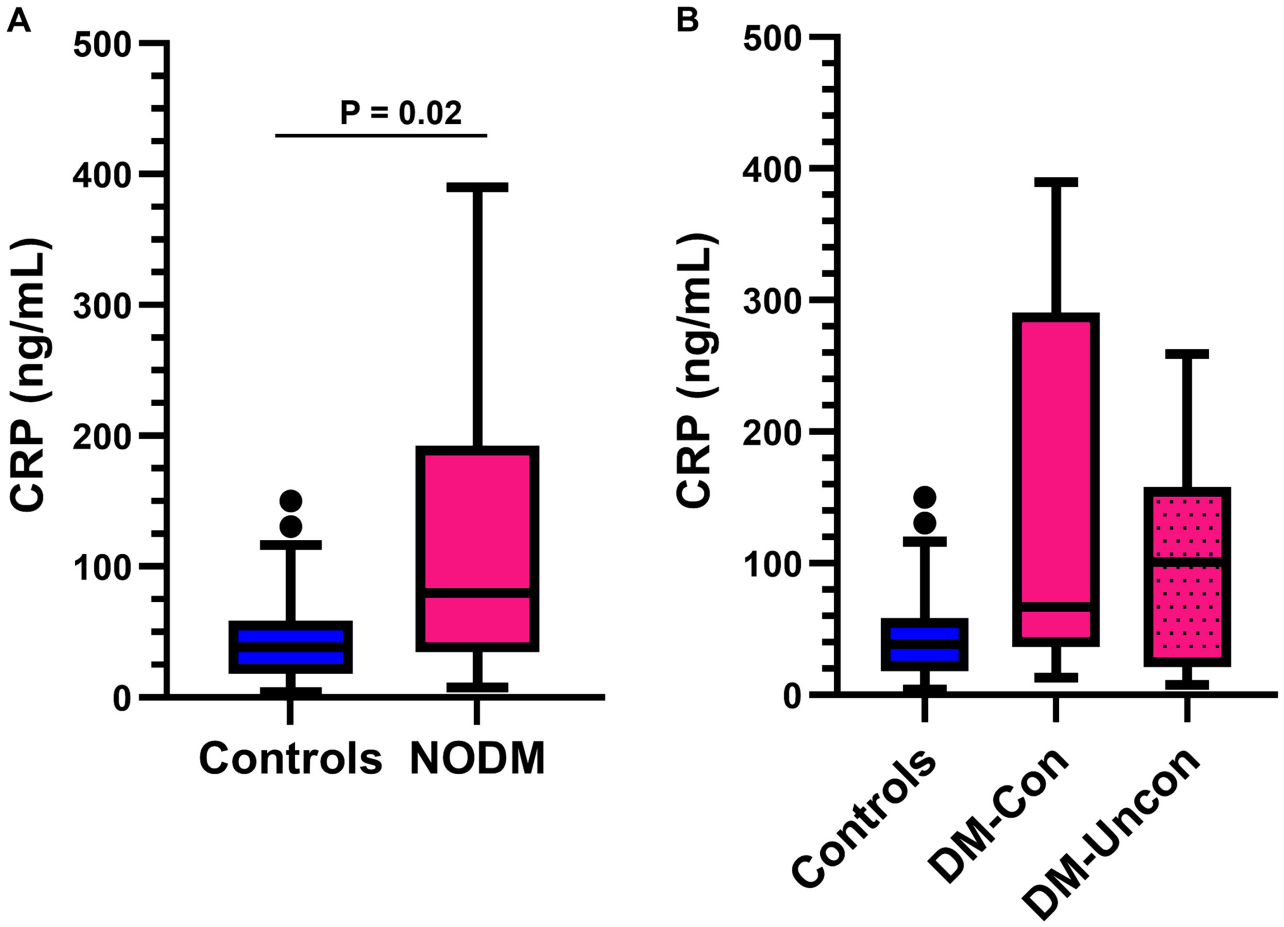
Box and whisker plots comparing serum C-reactive protein (CRP) between **(A)** dogs with naturally occurring diabetes mellitus (NODM) and controls, and between **(B)** diabetic dogs that were clinically controlled (DM-Con), uncontrolled (DM-Uncon), and controls. Line at median, bounds of box at the 25th and 75th percentile, whiskers at the upper and lower adjacent values (Tukey method), and dots at outliers beyond the adjacent values. **(A)** Diabetic dogs had higher serum CRP concentrations (median, IQR; 79.6 ng/mL, 157.4, n = 20) than controls (38.4 ng/mL, 40.4, *n* = 20, *p* = 0.02). **(B)** There was no difference between the medians for controls, DM-Con (66.8 ng/mL, 254.1, *n* = 10), and DM-Uncon (101.0 ng/mL, 137.1 *n* = 10, *p* = 0.06).

Diabetic dogs had higher serum CRP concentrations than controls (*p* = 0.02, Figure 2A). However, multigroup comparisons revealed no difference between diabetic dogs that were clinically controlled or uncontrolled, and controls (*p* = 0.06, Figure 2B).

### Phagocytic function of *E. coli*

**Figure 3:**
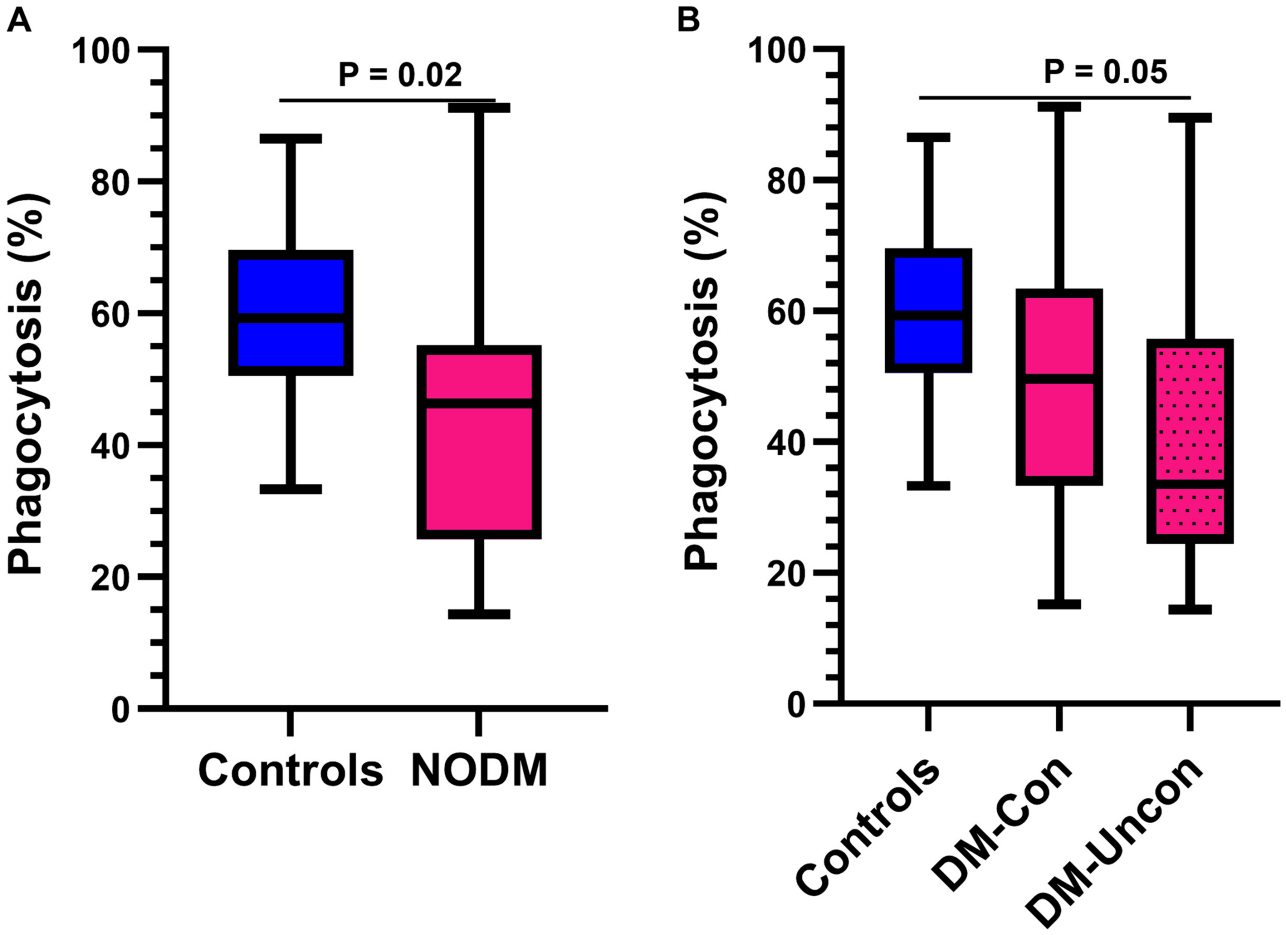
Box and whisker plots comparing percentage of granulocytes and monocytes performing phagocytosis of *Escherichia coli* (*E. coli*) between **(A)** dogs with naturally occurring diabetes mellitus (NODM) and controls, and between **(B)** diabetic dogs that were clinically controlled (DM-Con), uncontrolled (DM-Uncon), and controls. Line at median, bounds of box at the 25th and 75th percentile, whiskers at the upper and lower adjacent values (Tukey method), and dots at outliers beyond the adjacent values. **(A)** Diabetic dogs had a lower percentage of cells performing phagocytosis of *E. coli* (median, IQR; 46.3%, 29.5, *n* = 20) than controls (59.3%, 19.1, *n* = 20; *p* = 0.02). **(B)** There was a difference between the medians for phagocytic percentage for controls, DM-Con (49.7%, 30.2, *n* = 10), and DM-Uncon (33.5%, 31.3, *n* = 10, *p* = 0.04). Phagocytic percentage was lower in DM-Uncon compared to controls (*p* = 0.05). No differences were identified between DM-Con and controls (*p* = 0.39) or between DM-Con and DM-Uncon (*p* = 1.00).

Diabetic dogs had a lower percentage of GM that phagocytized opsonized-*E. coli* than controls (*p* = 0.02, Figure 3A) and differences were identified in multigroup comparisons (*p* = 0.04). Diabetic dogs that were clinically uncontrolled had a lower phagocytic percentage than controls (*p* = 0.05; Figure 3B).

**Figure 4:**
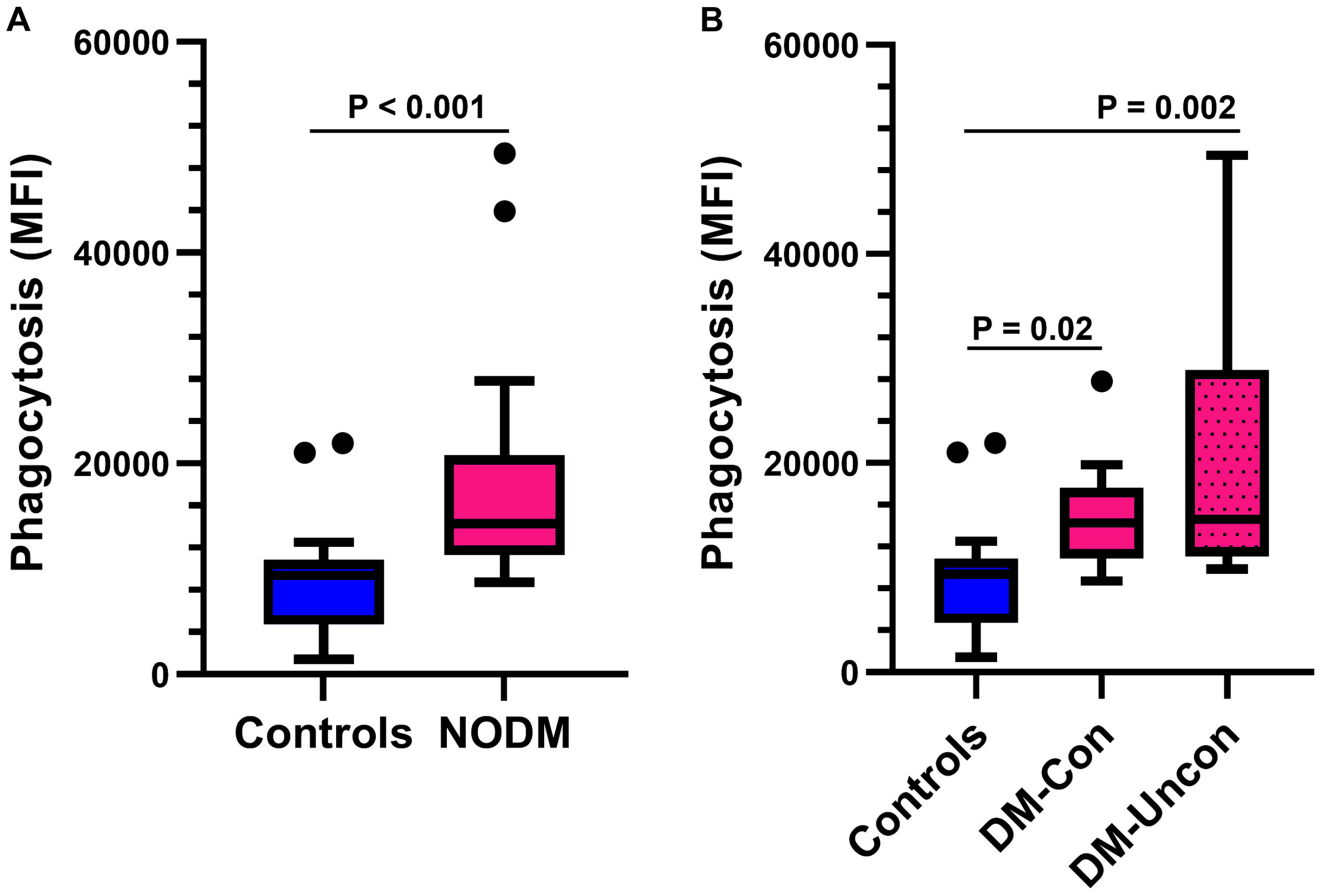
Box and whisker plots comparing the number of *Escherichia coli* (*E. coli*) phagocytized per cell between **(A)** dogs with naturally occurring diabetes mellitus (NODM) and controls, and between **(B)** diabetic dogs that were clinically controlled (DM-Con), uncontrolled (DM-Uncon), and controls. Line at median, bounds of box at the 25th and 75th percentile, whiskers at the upper and lower adjacent values (Tukey method), and dots at outliers beyond the adjacent values. **(A)** Diabetic dogs had a higher number of bacteria phagocytized per cell (median, IQR; 14,250 per cell, 9,475, *n* = 20) than controls (9392.6 per cell, 6129.9, *n* = 20, *p* < 0.001). **(B)** There was a difference between the medians for number of bacteria phagocytized per cell for controls, DM-Con (14,250 per cell, 6725.6, *n* = 10), and DM-Uncon (14,600 per cell, 17,825, *n* = 10, *p* < 0.001). The number of phagocytized bacteria per cell was higher in DM-Con (*p* = 0.02) and DM-Uncon (*p* = 0.002) compared to controls. No differences were identified between DM-Con and DM-Uncon (*p* = 0.50).

The number of phagocytized opsonized-*E. coli* per cell was higher in diabetic dogs compared with controls (*p* < 0.001, Figure 4A) and differences were also identified in multigroup comparisons (*p* < 0.001). Diabetic dogs that were clinically controlled (*p* = 0.02) and those that were uncontrolled (*p* = 0.002) had a higher number of phagocytized opsonized-*E. coli* per cell than controls (Figure 4B).

### Vitamin D metabolites

**Figure 5:**
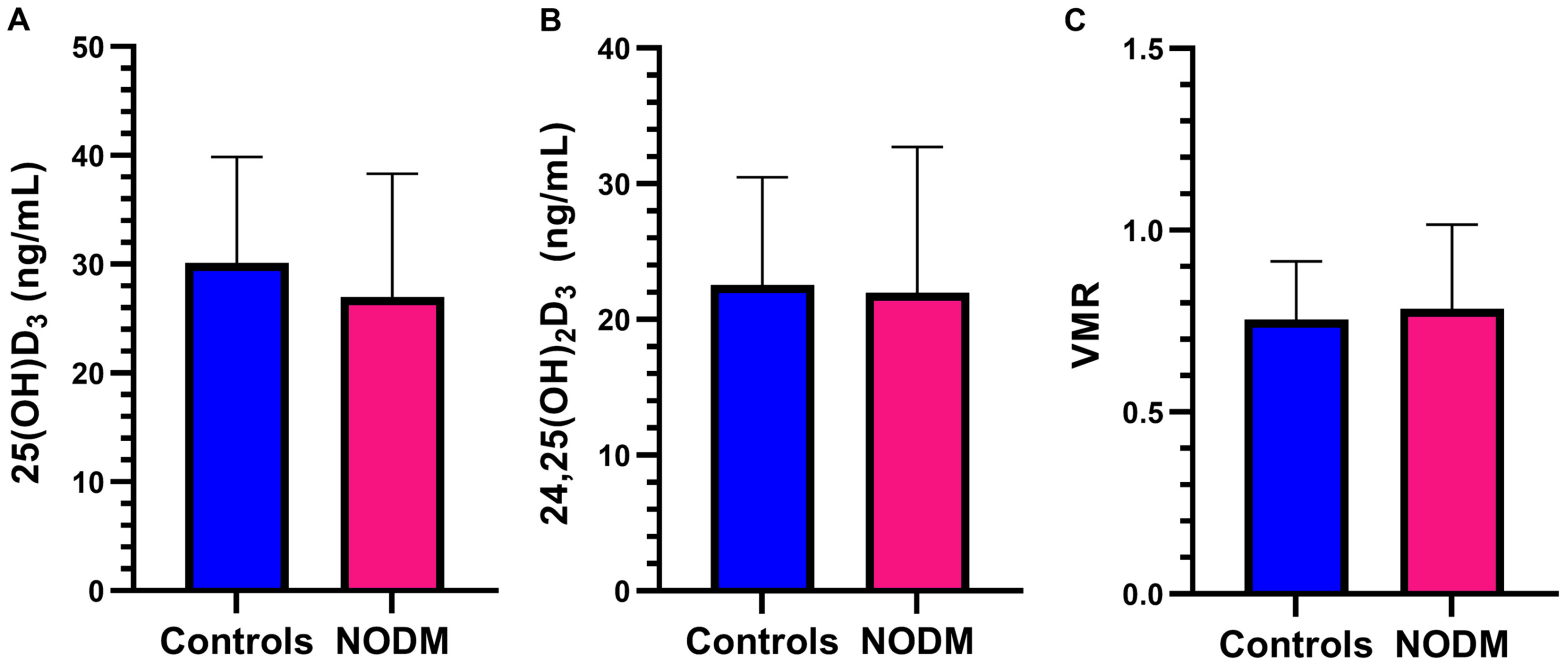
Bar graph comparing means of **(A)** 25-hydroxyvitamin (OH)D_3_, **(B)** 24,25-dihydroxyvitamin (OH)_2_D_3,_ and **(C)** Vitamin D metabolite ratio (VMR) between dogs with naturally occurring diabetes mellitus (NODM) and controls. Bar represents standard deviation. There were no differences in the means for 25(OH)D_3_ (NODM: 27 ng/mL, 11.3, *n* = 20; controls: 30.1 ng/mL, 9.7, *n* = 20, *p* = 0.35), 24,25(OH)_2_D_3_ (NODM: 22.4 ng/mL, 10.1, *n* = 19; controls: 22.6 ng/mL, 7.8, *n* = 20, *p* = 0.94), or VMR (NODM: 0.82, 0.20, *n* = 19; controls: 0.76, 0.16, *n* = 20, *p* = 0.31).

**Figure 6:**
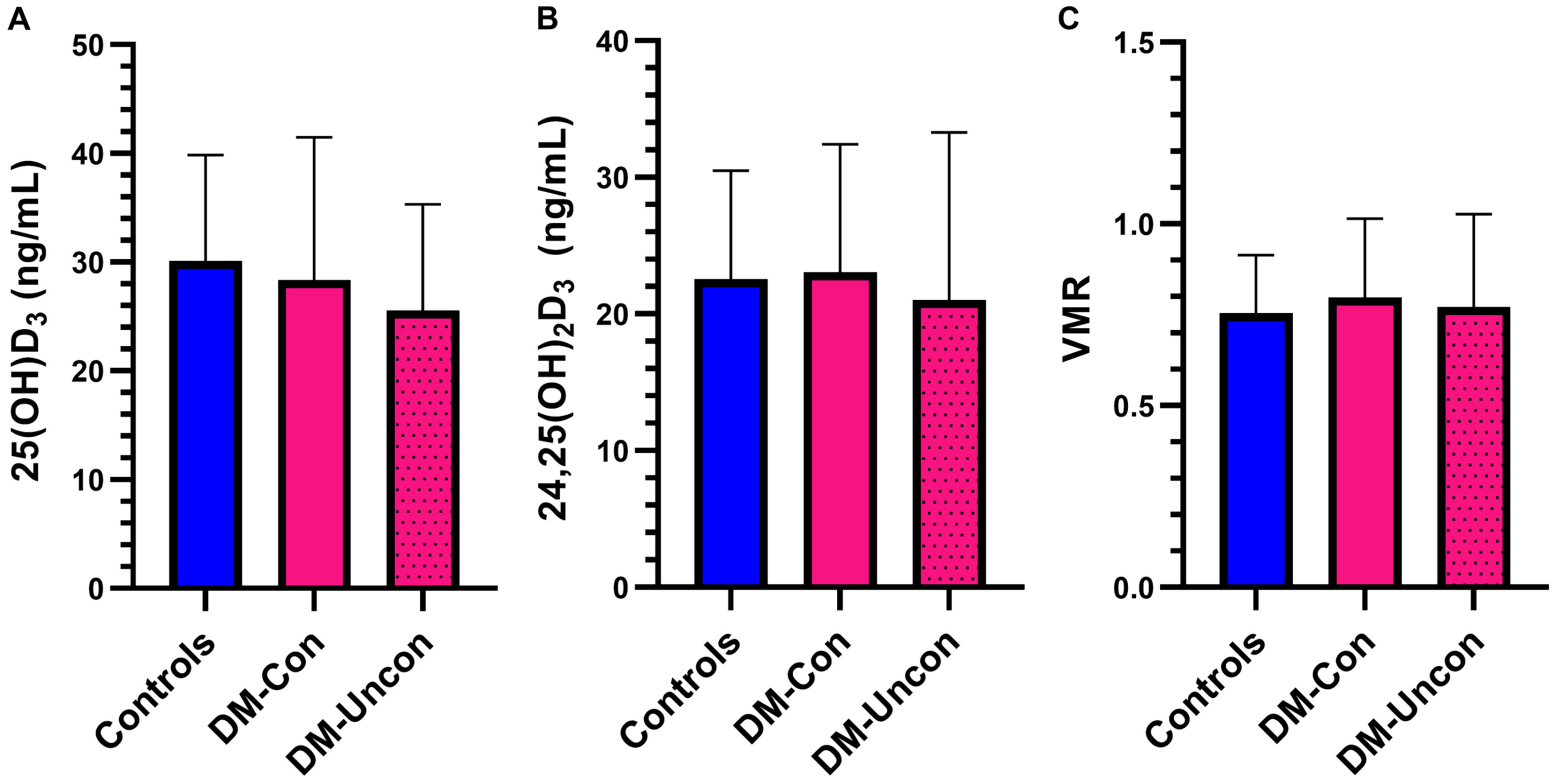
Bar graph comparing means of **(A)** 25-hydroxyvitamin (OH)D_3_, **(B)** 24,25-dihydroxyvitamin (OH)_2_D_3,_ and **(C)** Vitamin D metabolite ratio (VMR) between diabetic dogs that were clinically controlled (DM-Con), uncontrolled (DM-Uncon), and controls. Bar represents standard deviation. There was no difference between the means for 25(OH)D_3_ (DM-Con: 28.4 ng/mL, 13.1, *n* = 10; DM-Uncon: 25.6 ng/mL, 9.7, *n* = 10; controls: 30.1 ng/mL, 9.7, *n* = 20, *p* = 0.55), 24,25(OH)_2_D_3_ (DM-Con: 23.1 ng/mL, 9.4, *n* = 9; DM-Uncon: 21.8 ng/mL, 11.3, *n* = 10; controls: 22.6 ng/mL, 7.9, *n* = 20, *p* = 0.95) or VMR (DM-Con: 0.80, 0.22, *n* = 9; DM-Uncon: 0.83, 0.20, *n* = 10; controls: 0.76, 0.16, *n* = 20, *p* = 0.55).

There were no differences in two group or multigroup comparisons of serum 25(OH)D_3_, 24,25(OH)_2_D_3_, and VMR (*p* > 0.05; Figures 5, 6). The median serum 25(OH)D_3_ concentrations for the three control dogs with suspected mild osteoarthritis was 27.6 ng/mL (range, 24.2–38.2 ng/mL).

### Correlation analyses

First, we were interested in evaluating the relationships between vitamin D metabolites and various immunologic variables and fructosamine. No associations were identified between serum 25(OH)D_3_, 24,25(OH)_2_D_3_, or VMR with fructosamine, CRP, IL-8, or phagocytosis of *E. coli* (Table 2). There was a strong positive association between 25(OH)D_3_ and 24,25(OH)_2_D_3_ (rho = 0.78; *n* = 38, *p* < 0.001; Supplementary Figure S1).

**Table 2:** Association between 25-hydroxyvitamin (OH)D_3_, 24,25-dihydroxyvitamin (OH)_2_D_3_, and vitamin D metabolite ratio (VMR) with C-reactive protein (CRP), interleukin (IL)-8, fructosamine, and phagocytic function of *Escherichia coli* in 40 dogs that were either non-diabetic healthy controls (*n* = 20) or dogs with naturally occurring diabetes mellitus (*n* = 20).

Variable	Number	Rho	*p*-value
**25(OH)D**_**3**_			
Fructosamine (μmol/L)	40	−0.23	0.16
CRP (ng/mL)	40	−0.05	0.76
IL-8 (pg/mL)	40	−0.06	0.69
Phagocytosis (%)	40	0.24	0.13
Phagocytosis (MFI)	40	0.14	0.40
**24,25(OH)**_**2**_ **D**_**3**_			
Fructosamine (μmol/L)	39	−0.21	0.19
CRP (ng/mL)	39	0.01	0.94
IL-8 (pg/mL)	39	−0.16	0.34
Phagocytosis (%)	39	0.14	0.41
Phagocytosis (MFI)	39	0.18	0.28
**VMR**			
Fructosamine (μmol/L)	39	−0.003	0.98
CRP (ng/mL)	39	0.12	0.46
IL-8 (pg/mL)	39	−0.05	0.74
Phagocytosis (%)	39	−0.07	0.68
Phagocytosis (MFI)	39	0.06	0.72

MFI, mean fluorescence intensity.

Next, we aimed to determine whether there were any associations between serum fructosamine and various immunologic variables. There was a weak positive association between serum fructosamine and CRP concentrations (rho = 0.35, *p* = 0.03, *n* = 40; Table 3). A moderate positive association was identified between serum fructosamine and the number of phagocytized opsonized-*E. coli* per cell (rho = 0.45, *p* = 0.004, *n* = 40; Table 3). No associations were identified when just the diabetic dogs were included in these correlation tests (*p* > 0.05, *n* = 20; Supplementary Table S1).

**Table 3:** Association between serum fructosamine and C-reactive protein (CRP), interleukin (IL)-8, and phagocytic function of *Escherichia coli* in 40 dogs that were either non-diabetic healthy controls (*n* = 20) or dogs with naturally occurring diabetes mellitus (*n* = 20).

Variable	Number	Rho	*p*-value
CRP (ng/mL)	40	0.35	0.03
IL-8 (pg/mL)	40	0.23	0.16
Phagocytosis (%)	40	−0.23	0.16
Phagocytosis (MFI)	40	0.45	0.004

## Discussion

The inflammatory milieu, immunologic response, and role of vitamin D in people with T1DM has been well characterized but a relative void exists in dogs with NODM. Therefore, the goal of this prospective case–control study was to gather information to better understand cytokine homeostasis, phagocytic capacity of *E. coli*, vitamin D metabolites and the effect that clinical control has on these variables. We found that diabetic dogs had a higher proclivity for an inflammatory phenotype as seen with higher serum CRP concentrations compared to controls. Plasma cytokine concentrations were below the limit of detection in most dogs, regardless of group, except for IL-8. There was no difference in plasma IL-8 concentrations between all diabetic dogs compared to controls; however, diabetic dogs with clinically uncontrolled disease had higher levels compared with controls. Diabetic dogs exhibited abnormal phagocytosis and clinical status influenced these derangements. There were no two-group or multigroup differences in vitamin D metabolites nor were any associations with fructosamine, CRP, IL-8, or phagocytosis of *E. coli* identified. Lastly, positive associations were identified between fructosamine and CRP and the number of phagocytized bacteria per cell.

Serum CRP concentrations were higher in diabetic dogs compared to controls. The multigroup comparison revealed no differences in CRP concentrations with a *p*-value just outside of statistical significance (*p* = 0.06). Observationally, the medians for diabetic dogs that were uncontrolled (101 mg/L) and controlled (66.8 mg/L) were higher than non-diabetic controls (38.4 mg/L). Moreover, a mild positive significant association was detected between serum CRP and fructosamine concentrations. Collectively, these results suggest that otherwise healthy diabetic dogs have a pro-inflammatory phenotype with changes in severity occurring in tandem with fructosamine, a surrogate marker for glycemic control in dogs (49). Ours is the first study to evaluate the role of CRP in diabetic dogs and its association with diabetic management; however, this positive acute phase protein has been extensively studied in people with T1DM because of its association with glycemic control and diabetic complications (12, 16, 23, 24, 50–52). Importantly, the direction of relationship between serum CRP and fructosamine could not be ascertained in our study because of the observational design. It is equally plausible that hyperglycemia induced CRP secretion contributing to inflammation as it is that increased CRP secondary to an unrelated source of inflammation-induced insulin resistance that affected glucose metabolism (53–56). Prospective studies with longitudinal assessment of serum CRP and glycemic control are needed to better define causality in this association.

Plasma IL-8 concentrations were higher in all diabetic dogs compared to controls, but this difference did not reach statistical significance (*p* = 0.09). These results contrast with findings from the only other study to have investigated blood IL-8 concentrations in diabetic dogs (27). The aforementioned study using the same canine specific multiplex bead-based assay to quantify cytokine concentrations found that diabetic dogs had higher basal serum IL-8 concentrations (median, range; 2,590 pg./mL, 1,410–3,580 pg./mL, *n* = 9) compared with healthy controls (1,070 pg./mL, 828–1910 pg./mL, *n* = 9) (27). One possible explanation for the conflicting results is that the reported IL-8 concentrations for diabetic dogs were 5 times higher in the O’Neill et al. study compared to our diabetic cohort. The included diabetic dogs in that study were enrolled at a routine follow-up visit and the presence of concurrent disease was not exclusionary. Therefore, it is reasonable to suspect that some of the dogs had comorbid disorders that influenced serum IL-8 concentrations. We only included otherwise healthy diabetic dogs in our study to avoid confounding effects of other disorders on immunologic findings. Alternatively, or in addition, our study may have been underpowered to detect a difference if one existed (i.e., Type 2 error). The latter theory seems reasonable as elevated basal blood IL-8 concentrations in people with T1DM is one of the most consistently reported cytokine derangements (57–61).

Diabetic dogs with uncontrolled clinical disease had higher basal plasma IL-8 concentrations than non-diabetic controls. However, there were no differences identified between diabetic dogs with controlled clinical signs and either uncontrolled diabetics or non-diabetic controls. Collectively, these results suggests that dogs with poorly regulated diabetes are more likely to have higher basal circulating levels of IL-8. This theory is supported by results from one study that showed basal serum IL-8 concentrations were higher in children with T1DM that had poor glycemic control compared to those with favorable control and there was a positive correlation between IL-8 concentrations and HbA1c (60). We cannot determine based on the design of our study whether elevated basal plasma IL-8 concentrations were a cause, consequence, or a combination thereof, for poorly regulated diabetic clinical control. Persistent uncontrolled hyperglycemia can directly increase production of IL-8. This has been highlighted in previous work in which hyperglycemia induced transcription of the gene for IL-8 in endothelial cells and monocytes (62, 63). However, increases in IL-8 can cause insulin resistance and thus worsen hyperglycemia via inhibition of insulin-induced Akt phosphorylation (64). Overall, IL-8 is one of the most important molecules involved with initiation and amplification of the acute inflammatory response and has been linked with development of diabetes related complications in people (65, 66). The combination of our findings in dogs and increased risk for diabetes-related complications in people with elevated IL-8 support the need for further investigation into the role of this chemokine in diabetic dogs.

Basal plasma concentrations of TNF-α, IL-6, and IL-10 were below the limits of detection in most dogs, regardless of group. These results are in contrast with common findings in diabetic people but are similar to results found in the diabetic dog study by O’Neill et al. (15, 16, 27, 58). One potential explanation for the lack of quantifiable cytokine detection is that diabetes mellitus is a dynamic disorder with expected changes in the inflammatory milieu from the time of diagnosis to various time-points after institution of insulin therapy. One study found that only children with newly diagnosed (i.e., < 1 year) T1DM had increased basal serum TNF-α and IL-6 concentrations (58). Another study found that mitogen-stimulated peripheral blood mononuclear cell production of TNF-α in supernatant decreased over time in patients as glycemic control improved (19). We evaluated plasma cytokine concentrations on a single occasion a median of 302 days after dogs were diagnosed with NODM. Therefore, it is possible we missed elevations in these cytokines because of when samples were procured. Another point to consider is that the multiplex assay used in this study may have been relatively insensitive to identify small changes in cytokine concentrations in dogs. While this multiplex assay is widely used in dogs, some cytokine concentrations are commonly below the limits of detection independent of health status (27, 67–71).

Leukocytes from diabetic dogs exhibited abnormal phagocytic activity of *E. coli* characterized by a decreased percentage of cells performing phagocytosis with a concomitant increase in number of bacteria phagocytized per cell compared to non-diabetic controls. The cumulative effect of these findings on the overall phagocytic capacity of *E. coli* in diabetic dogs is unknown. One possible explanation for these results is that there is a global reduction in leukocytes performing phagocytosis and the remaining available cells must increase activity to compensate. There have been several studies over the last 40 years that have highlighted impaired leukocyte phagocytic activity of various substrates in people with T1DM and animal models (20, 72–78). Studies in rabbits and mice demonstrate a clear causal role for hyperglycemia decreasing leukocyte phagocytosis that is restored with insulin (79–81). Diabetes-associated impaired phagocytosis is multifactorial with several purported possible mechanisms that include intracellular accumulation of sorbitol, decreased phosphofructokinase activity, and dysregulated metabolism of arachidonic acid (20). The positive association between fructosamine and the number of bacteria phagocytized per cell was an unexpected finding in our study. Explanations for this finding are beyond the scope of this study but warrant further investigation.

There were no differences in vitamin D metabolites between diabetic dogs and controls, nor were differences identified based on the level of diabetic clinical control. These findings are in contrast with our hypothesis and commonly reported results from people with T1DM (29–31). The modest size of the sample population in our study may have contributed to type 2 error as well as limited genetic variability. Studies in human patients with T1DM have identified genetic polymorphisms in vitamin D handling proteins that can affect vitamin D blood concentrations, cellular availability, and metabolism (82–84). Therefore, it is possible a much larger sample population would have accounted for this potential genetic variability. It must also be considered that the lack of differences in vitamin D metabolites in our study is real and may represent a species difference. We did not find associations between vitamin D metabolites and any immune function variables in our study. A potential explanation for this is the low proportion of dogs with severely low serum 25(OH)D concentrations (< 25–30 ng/mL) (85). The risk for various disorders and associative immune dysregulation is higher in people with vitamin D deficiency (i.e., serum/plasma 25(OH)D of <25–30 ng/mL) (86, 87).

Our study had several limitations that must be considered. The diabetic dogs in this study were determined to be otherwise healthy based on a collective interpretation of history, physical examination, complete blood count, biochemical panel, and urinalysis by a board-certified small animal internist. It is possible some diabetic dogs included in this study had an occult disorder that affected assessment of inflammatory biomarkers and immune function testing. Commercially available dog foods have varying vitamin D content that may influence serum 25(OH)D concentrations (85). However, there is a curvilinear phenomenon in the association between vitamin D intake and blood 25(OH)D concentrations in dogs. As vitamin D intake increases, the response in rising 25(OH)D_3_ diminishes. At the low end of vitamin D intake, circulating 25(OH)D_3_ rises sharply with minor increases in vitamin D intake (88). Commercial dog food is typically adequately supplied with vitamin D. Therefore, otherwise healthy dogs offered these dog foods are unlikely to be vitamin D deficient. We chose to allow dogs to continue their typical maintenance diets rather than switch the entire cohort to a single diet for two primary reasons. First, changing the offered diet would have likely affected diabetic regulation in some dogs and possibly caused adverse food reactions in others. Secondly, we wanted our cohort to be representative of a typical population of diabetic and control dogs. Regardless, the lack of difference in vitamin D metabolites indicates that diet had a minimal influence in our study. Diabetic dogs were grouped based on the level of clinical control, which does not always parallel glycemic control. Several recent studies have highlighted limitations of relying on biomarkers such as fructosamine to determine glycemic control in dogs (89, 90). Studies that investigate the role of glycemic control on inflammatory biomarkers and immune function may be better served grouping dogs based on a combination of clinical status and data from continuous glucose monitors. Assessment of phagocytosis was limited to opsonized-*E. coli* only and thus our results cannot be extrapolated as an overall indictment of phagocytic capacity in diabetic dogs. Future studies including various substrates to assess phagocytosis would be useful to attain a more comprehensive assessment. We were intentional in our investigation of CRP, TNF-α, IL-6, IL-8, and IL-10 because these biomarkers are most commonly implicated in diabetic people (12, 15–17, 19, 24, 36, 91, 92). However, a more comprehensive profile including more cytokines and acute phase proteins may provide a more global picture of the inflammatory microenvironment in diabetic dogs. Three control dogs were suspected to have mild osteoarthritis. Hypovitaminosis D is a relatively common finding in people with osteoarthritis but has not been thoroughly investigated in dogs (93). Therefore, it is possible that osteoarthritis in these dogs may have had a mild effect on serum 25(OH)D_3_ concentrations but was not believed to have substantially impacted statistical analyses because the median serum 25(OH)D_3_ concentration in these three control dogs were comparable to the mean results for both controls and diabetics. Lastly, we collected samples for our experiments at a single time-point after initiation of insulin therapy. A longitudinal study design starting at the time of diagnosis (before insulin therapy) with serial sampling through the time that adequate glycemic control is achieved would provide integral information regarding immunologic changes over time while also more confidently concluding causality.

## Conclusion

In conclusion, our study demonstrates that diabetic dogs have an inflammatory phenotype and phagocytic dysregulation of opsonized-*E. coli*. Uncontrolled clinical disease in diabetic dogs may worsen the magnitude of inflammation and phagocytic derangements. Lastly, higher serum fructosamine concentrations were correlated with increasing CRP concentrations and number of bacteria phagocytized per cell but causality was not determined.

## Data Availability

The raw data supporting the conclusions of this article will be made available by the authors, without undue reservation.
