# Supplementary figures and images for: Evaluation of serum vitamin D metabolites, phagocytosis, and biomarkers of inflammation in dogs with naturally occurring diabetes mellitus
Source: Front Vet Sci. 2024 Aug 21;11:1441993. doi: 10.3389/fvets.2024.1441993 (PMC11371797; doi:10.3389/fvets.2024.1441993)

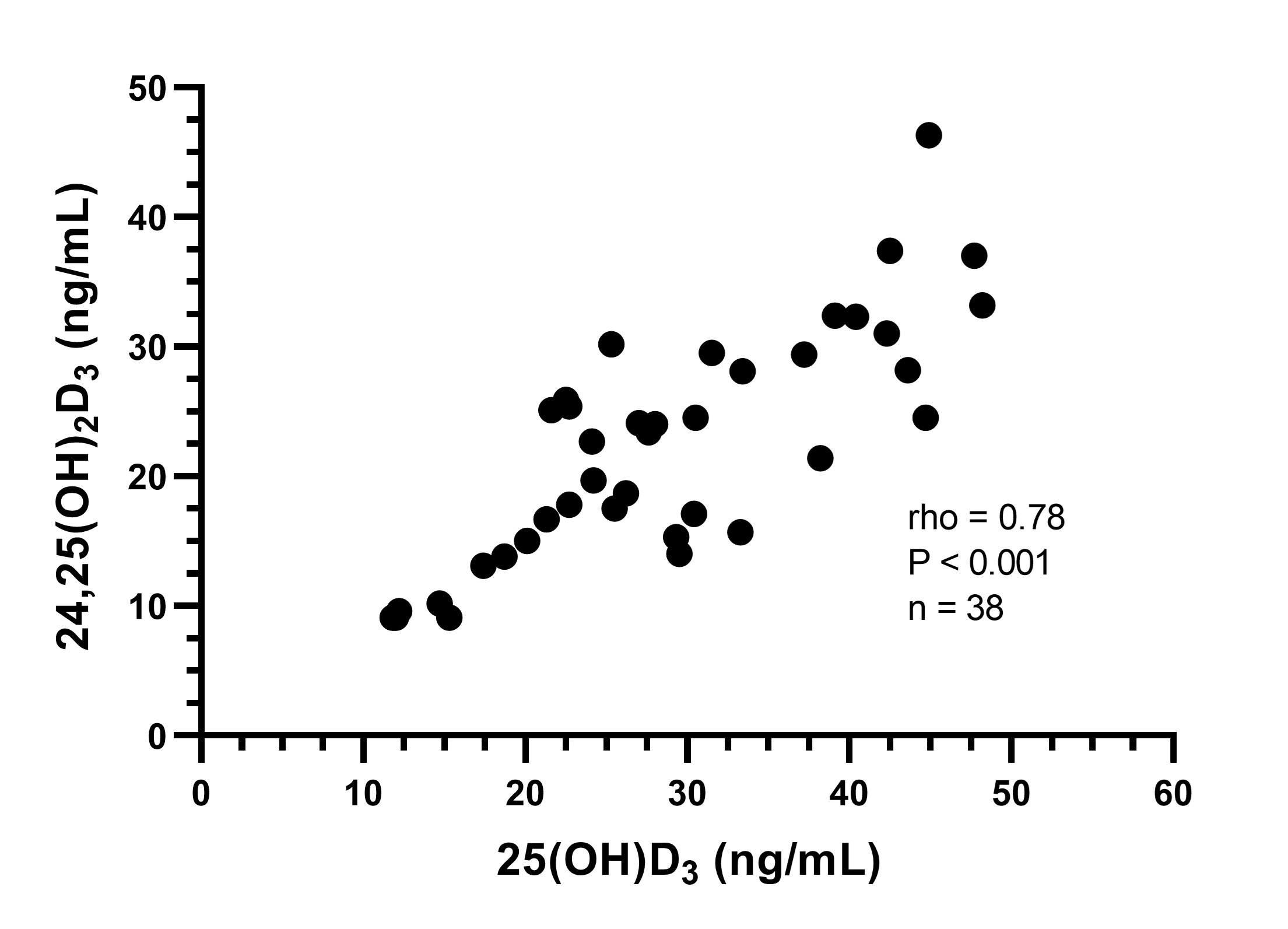

Supplement: Supplementary file 1 [file Image1.JPEG]
