# Supplementary material for: Evaluation of serum vitamin D metabolites, phagocytosis, and biomarkers of inflammation in dogs with naturally occurring diabetes mellitus
Source: Front Vet Sci. 2024 Aug 21;11:1441993. doi: 10.3389/fvets.2024.1441993 (PMC11371797; doi:10.3389/fvets.2024.1441993)
Supplement: Supplementary file 2 [file Table1.DOCX]

Supplemental Table 1. Association between serum fructosamine and C-reactive protein (CRP), interleukin (IL)-8, and phagocytic function of Escherichia coli in 20 dogs with naturally occurring diabetes mellitus.

| **Variable** | **Number** | **Rho** | **P-value** |
| --- | --- | --- | --- |
| CRP (ng/mL) | 20 | 0.04 | 0.85 |
| IL-8 (pg/mL) | 20 | - 0.03 | 0.90 |
| Phagocytosis (%) | 20 | 0.21 | 0.37 |
| Phagocytosis (MFI) | 20 | - 0.01 | 0.95 |
