# Supplementary material for: Evaluation of serum vitamin D metabolites, phagocytosis, and biomarkers of inflammation in dogs with naturally occurring diabetes mellitus
Source: Front Vet Sci. 2024 Aug 21;11:1441993. doi: 10.3389/fvets.2024.1441993 (PMC11371797; doi:10.3389/fvets.2024.1441993)
Supplement: Supplementary file 3 [file Table2.DOCX]

**Supplemental Material**

*Non-diabetic healthy control dogs were fed the following commercial diets*: Royal Canin Size Health Nutrition Small Adult kibble (n = 3), Purina Beneful IncrediBites Small Breed Dry kibble (n = 2), Hill’s Metabolic Weight Loss & Maintenance kibble (n =2), Royal Canin Size Health Nutrition Medium Adult kibble, Hill’s Science Diet Adult 7+ Senior kibble, Blue Buffalo Chicken and Brown Rice kibble, Grandma Mae’s Grain Free Multi-Protein kibble and can, Purina Pro Plan Chicken and Rice kibble, Pedigree Dry Dog Food Small Dog Grilled Steak and Vegetable Flavor kibble, Nutro Natural Choice Senior Dog kibble, Hill’s Science Diet Adult Small Breed kibble, Hill’s Science Diet Adult Large Breed kibble, Kirkland Signature Healthy Weight Formula kibble, Rachael Ray Nutrish Real Chicken & Veggies Recipe Dry kibble, Nutro Natural Choice Adult Chicken & Brown Rice recipe kibble, Purina Beneful Healthy Weight kibble.

*Diabetic dogs were fed the following commercial diets*: Hill’s W/D Multi-Benefit kibble (n = 5), Royal Canin Glycobalance kibble and can, Hill’s Science Diet kibble and canned, Iams kibble, Royal Canin Glycobalance kibble, Hill’s W/D Multi-Benefit kibble and canned, Hill’s Science Diet Adult Light Chicken Meal & Barely Dry kibble, Rachael Ray Nutrish Real Chicken & Veggies Recipe Dry kibble, Freshpet, Dr. Harvey’s Beef & Garden Veggies, Nutro Natural Choice Adult Salmon & Lentils Recipe kibble, Hill’s I/D Digestive Care Chicken kibble, Pedigree Adult Complete Nutrition, Purina ONE +Plus Vibrant Maturity Adult 7+ Formula Dry kibble, The Honest Kitchen Whole Grain Chicken Recipe Dehydrated kibble, Halo Holistic Vegan Plant-Based with Kelp Recipe kibble
